# Brains swinging in concert: cortical phase synchronization while playing guitar

**DOI:** 10.1186/1471-2202-10-22

**Published:** 2009-03-17

**Authors:** Ulman Lindenberger, Shu-Chen Li, Walter Gruber, Viktor Müller

**Affiliations:** 1 Center for Lifespan Psychology, Max Planck Institute for Human Development, Lentzeallee 94, 14195 Berlin, Germany; 2 Department of Physiological Psychology, University of Salzburg, 5020 Salzburg, Austria

## Abstract

**Background:**

Brains interact with the world through actions that are implemented by sensory and motor processes. A substantial part of these interactions consists in synchronized goal-directed actions involving two or more individuals. Hyperscanning techniques for assessing fMRI simultaneously from two individuals have been developed. However, EEG recordings that permit the assessment of synchronized neuronal activities at much higher levels of temporal resolution have not yet been simultaneously assessed in multiple individuals and analyzed in the time-frequency domain. In this study, we simultaneously recorded EEG from the brains of each of eight pairs of guitarists playing a short melody together to explore the extent and the functional significance of synchronized cortical activity in the course of interpersonally coordinated actions.

**Results:**

By applying synchronization algorithms to intra- and interbrain analyses, we found that phase synchronization both within and between brains increased significantly during the periods of (i) preparatory metronome tempo setting and (ii) coordinated play onset. Phase alignment extracted from within-brain dynamics was related to behavioral play onset asynchrony between guitarists.

**Conclusion:**

Our findings show that interpersonally coordinated actions are preceded and accompanied by between-brain oscillatory couplings. Presumably, these couplings reflect similarities in the temporal properties of the individuals' percepts and actions. Whether between-brain oscillatory couplings play a causal role in initiating and maintaining interpersonal action coordination needs to be clarified by further research.

## Background

Brain activities supporting human social interactions have recently become an important topic of scientific inquiry [1-6]. Considerable research indicates that synchronized neuronal activity in perception and action [7-10] and oscillatory couplings between cortical and muscle activities during voluntary movement [11,12] are among the mechanisms supporting brain-body-world interactions [6,13-15]. A substantial part of these interactions consists in synchronized goal-directed actions involving two or more individuals [1-5]. In everyday life, people often need to coordinate their actions with that of others. Some common examples are walking with someone at a set pace, playing collective sports or fighting [16], dancing [16,17], playing music in a duet or group [18], and a wide range of social bonding behaviors (e.g., eye-gaze coordination between mother and infant or between partners).

Little, if anything, is known about brain mechanisms implementing interpersonally coordinated behavior. These mechanisms most likely will have to meet two constraints: (a) they need to be sufficiently fast to permit fluidity in interpersonal action coordination; (b) they need to integrate and regulate sensory, motor, and brain activity to generate and sustain action coordination between two or more persons. Synchronous oscillatory brain activities appear to meet both criteria. First, brain oscillations bind spatially distributed but functionally related information at the level of individual neurons, cell assemblies, and cortical areas [6-10]. Onset times and frequency ranges of coherent or synchronized oscillations are sufficiently fast [19] to permit, in principle, the speed and precision of information exchange required by interpersonal action coordination. Second, coherent oscillations support both perception [2,12,20] and motor performance [11,12,21]. Specifically, coherence between neuronal activities measured by magnetoencephalography (MEG) or electroencephalography (EEG) and muscle activity measured by electromyography (EMG) has been observed during voluntary movement control [11,12]. Thus, coherent oscillations between brains may support interpersonally coordinated behavior through reciprocal sensory and motor feedbacks. In two interacting individuals adjusting their activity patterns through reciprocal sensory and motor pathways, synchronous brain activity can arise through temporally adjusted activity modulation [22-24] and does not need a substrate in form of connected neurons. Such interbrain synchronization could represent and also support interpersonal action coordination and social interaction.

Recently, applying high-resolution spectral analysis to electrical brain activity of two persons measured simultaneously during visually mediated social interaction [25], it was found that power increase of the phi_1 _rhythm was associated with independent behavior, whereas power increase of the phi_2 _rhythm was related to coordinated behavior. A possible mechanism is that phi_1 _expresses the inhibition of the human mirror neuron system and phi_2 _its enhancement. Both of these oscillatory components reflect asymmetric spectral power between the interhemispheric pairs of electrodes in rows 3 and 4 of the 10%-montage. These two rhythms are in the frequency range between 9.2 and 11.5 Hz and were located above the right centro-parietal cortex. At the same time, the authors observed a depression in occipital alpha and mu rhythms during social interaction regardless of whether the behavior was coordinated or not. However, the authors did not directly examine the role of interbrain synchronization in coordinated action.

In the current study, we investigated whether the phase synchronization of brain oscillations within and between the brains is enhanced in pairs of guitarists during the preparatory period of metronome tempo setting and while playing a melody together. Synchronization at frontal and central electrode sites may indicate coordinated firing of neuronal assemblies located in the motor and somatosensory cortices, which control and coordinate motor activity and are activated during music production [26]. Furthermore, there is evidence to suggest that neural activity in medial prefrontal cortex is selectively enhanced during theory of mind tasks and the sensory representation of others [2,3,27,28]. In accordance with these findings, we expected that action-related within-brain synchronization and between-brain coherence would be most pronounced over fronto-central sites. Activation and synchronization at temporal and medial parietal brain regions were expected as well because these regions have been shown to be involved in music production [26] and coordinated behavior [25]. Based on findings in the literature that are related to coordinated behavior, we decided to restrict our analyses to frequency bands below 20 Hz. Oscillatory components in the alpha frequency range between 7.5 and 13 Hz have been found during visually mediated social coordination [25]. Moreover, changes in the P3 ERP component, which probably reflect low-frequency oscillations (e.g., in the delta range), have been observed in the context of interpersonally shared task representations [29]. In addition, low-frequency oscillations are involved in limb and hand movements [11,12,30-32], and in sensorimotor integration [33]. In sum, the frequency range up to 20 Hz seemes to be involved in interpersonal coordination and sensorimotor interaction, which are both important for interpersonal action coordination.

## Results

By simultaneously recording the EEG of two people, we measured brain electrophysiological activities from eight pairs of guitarists while playing a short melody together over about 60 trials (a video recording of a pair of guitarists playing together for a few trials with the corresponding EEG can be found in the Additional file 1 available online). The melody the guitarists played was taken from the first six measures of a modern jazz-fusion piece, "Fusion #1," composed by Alexander Buck (born 1979). The piece has the time signature of four quarter notes per measure, and was played in E minor. At the beginning of each trial, at least four metronome beats were played through a loudspeaker to both guitarists of each pair. The metronome frequencies, which were chosen by the different pairs of guitarists, ranged from 1.3 Hz to 2 Hz (see Figure S1 in the Additional file 2 for different time intervals between the beats). Thereafter, the lead guitarist (A) gave a sign to start, and then the duo started to play the melody together in unison. Synchronous brain activities within and between brains were investigated and analyzed by the Phase Locking Index (PLI) and Interbrain Phase Coherence (IPC), respectively. PLI reflects invariance of phases across trials measured from single electrodes within a brain in the time-frequency domain. IPC represents the degree of constancy in phase differences across trials between two electrodes measured from two brains simultaneously (see Methods). We analyzed 3s-sequences (1s before and 2s after event onset) that were time locked either (a) to the second metronome beat or (b) to the start of guitar playing by guitarist A (play onset). Using complex Gabor transformations, we calculated PLI and IPC for frequencies up to 20 Hz with a frequency resolution of 0.33 Hz. With respect to within-brain analyses, PLI values were firstly computed for 16 electrodes. Results from six the fronto-central electrodes (F3, Fz, F4, C3, Cz, and C4) were averaged in the time-frequency domain and are reported below. With respect to analyses of interbrain coherence (i.e., coherence between the brains of the two guitarists of each pair), IPC values were first computed from all possible pairwise connections (16 × 16) across the 16 electrodes selected for within-brain analyses. The same six fronto-central electrode pairs used for within-brain analyses were averaged in the time-frequency domain and reported below. Across all frequency bins, the statistical significance of observed PLI and IPC values was estimated relative to baselines that were defined to be (a) within the 300 ms window elapsing between two metronome beats, or (b) within the 300 ms window preceding playing onset, respectively. Mean PLI or IPC values three standard deviations above baseline were considered as statistically significant (*p *< 0.01). Values below this level are not presented in the time-frequency diagrams. Table 1 reports averages of the maximum PLI and IPC values for the 16 electrodes averaged across the 8 pairs of guitarists. In addition, maximum PLI and IPC values for each of the 16 electrodes and for each pair of guitarists are presented as radar plots in Additional file 2 (Figure S2).

**Table 1:** Mean (M) and standard deviation (SD) of PLI (Phase Locking Index) and IPC (Interbrain Phase Coherence) maxima across the 8 pairs of guitarists during the preparatory period of metronome setting and during the period of guitar playing separately for the 16 electrodes.

Measures	Electrodes																
		F7	F3	Fz	F4	F8	T7	C3	Cz	C4	T8	P7	P3	Pz	P4	P8	Oz
Metronome																	
PLI-A	M	.59	.64	.63	.63	.60	.58	.68	.65	.66	.65	.37	.50	.50	.48	.45	.34
	SD	.07	.08	.08	.07	.06	.12	.10	.09	.10	.14	.04	.07	.08	.08	.10	.06
PLI-B	M	.55	.60	.59	.62	.56	.47	.62	.60	.63	.52	.40	.48	.49	.47	.37	.36
	SD	.12	.11	.11	.12	.11	.10	.09	.09	.10	.09	.08	.11	.12	.12	.10	.06
IPC-AB	M	.44	.44	.42	.43	.43	.38	.46	.44	.45	.37	.37	.40	.40	.40	.35	.33
	SD	.06	.06	.08	.08	.07	.07	.06	.08	.07	.04	.08	.10	.09	.07	.09	.06
IPC-BA	M	.41	.45	.43	.45	.37	.43	.47	.44	.46	.43	.31	.36	.37	.38	.35	.31
	SD	.08	.09	.08	.07	.06	.09	.12	.08	.09	.10	.07	.08	.06	.05	.04	.04
																	
Play onset																	
PLI-A	M	.57	.63	.61	.60	.53	.49	.57	.57	.58	.52	.45	.51	.50	.53	.52	.44
	SD	.07	.06	.07	.06	.09	.11	.13	.10	.13	.12	.06	.13	.12	.15	.18	.14
PLI-B	M	.52	.59	.59	.57	.52	.55	.61	.61	.61	.52	.44	.49	.51	.54	.44	.43
	SD	.05	.08	.10	.09	.10	.11	.11	.11	.09	.16	.08	.14	.15	.12	.09	.12
IPC-AB	M	.41	.44	.43	.42	.39	.39	.46	.46	.45	.38	.36	.39	.39	.40	.38	.36
	SD	.09	.12	.13	.12	.11	.07	.10	.11	.10	.12	.08	.05	.05	.07	.10	.06
IPC-BA	M	.45	.48	.47	.47	.38	.40	.46	.46	.46	.37	.32	.38	.40	.42	.40	.32
	SD	.12	.09	.08	.11	.11	.09	.11	.11	.10	.11	.05	.11	.10	.11	.11	.10

PLI = Phase Locking Index; IPC = Interbrain Phase Coherence; A = Guitarist A; B = Guitarist B; AB = Cz electrode of guitarist A to the other electrode of guitarist B; BA = Cz electrode of guitarist B to the other electrode of guitarist A; M = Mean; SD = Standard Deviation.

### Synchronization during the preparatory period

**Figure 1:**
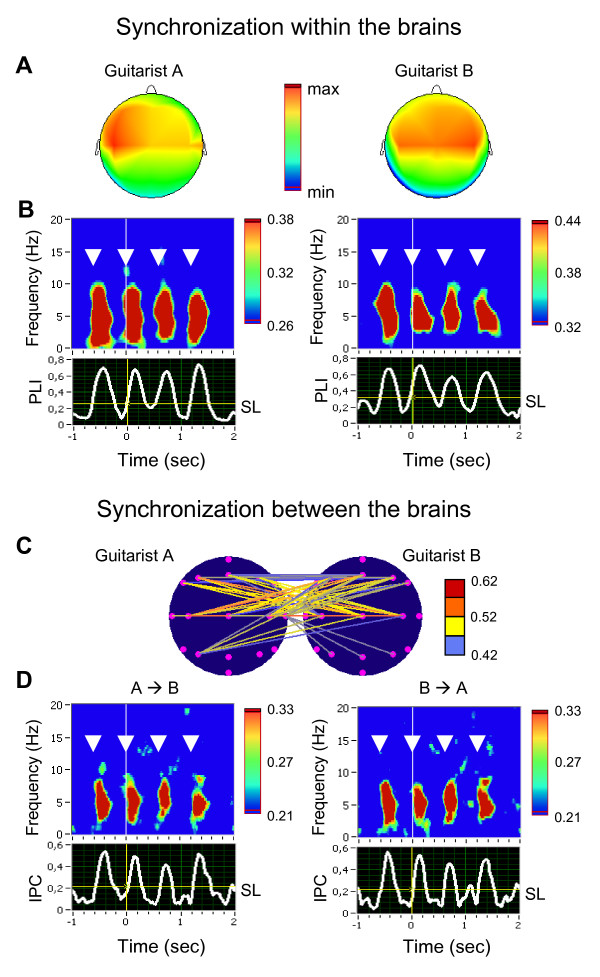
**Phase synchronization within and between brains during the preparatory period of metronome tempo setting**. (A) Topological distributions of PLI in a representative pair of guitarists, A and B, at the theta frequency (4.95 Hz) 140 ms after stimulus-onset (second metronome beat). Fronto-central maxima of PLI are shown. (B) Time-frequency diagrams of average PLI for guitarist A and B separately. PLI was averaged across six fronto-central electrodes. Only significant PLI-values (*p *< 0.01) are highlighted. Time zero is time locked to the second metronome beat. Metronome beats are shown by white arrows. The time course of PLI values at the theta frequency (4.95 Hz) is depicted below the time-frequency diagram. (C) Interbrain synchronization between the two guitarists measured by IPC at the theta frequency (4.95 Hz) 140 ms after stimulus onset. Colored lines indicate synchrony between electrode pairs of the two guitarists, corresponding to significant interbrain synchronization. Only IPC values higher than 0.41 are highlighted. (D) Time-frequency diagram of the average IPC averaged across six electrode pairs. In the left diagram (A -> B), the selected electrode pairs represent phase coherence between one electrode of guitarist A (Cz) to the six fronto-central electrodes of guitarist B. Conversely, the right diagram (B -> A) refers to one electrode of guitarist B and the six fronto-central electrodes of guitarist A. Only significant IPC-values (*p *< 0.01) are highlighted. The time course of IPC values at the theta frequency (4.95 Hz) is depicted below the time-frequency diagram. SL = significance level.

Synchronization within the brains as measured by PLI during the preparatory period of metronome tempo setting was highest at fronto-central sites (Figure 1A). Averaged PLI values from the six fronto-central electrodes (F3, Fz, F4, C3, Cz, and C4) were calculated in the time-frequency domain for each frequency bin and time lag. Based on the averaged PLI, synchronization within the brains was particularly high in the frequency range between 2 and 10 Hz with the maximum in the theta frequency band (3–7 Hz). This effect was strictly related to the onset of the metronome beats (Figure 1B) and was found practically in all participants (Figure S1). As for synchronization between brains as measured by IPC, coherence was also strongest for fronto-central connections (Figure 1C). Averaged IPC values from the Cz electrode of guitarist A to the six fronto-central electrodes (F3, Fz, F4, C3, Cz, and C4) of guitarist B and vice visa were thus calculated. Based on the averaged IPC, between-brain synchronization was most clearly observable in the frequency range between 3 and 8 Hz, with the maximum being around 5 Hz (Figure 1D). Interbrain phase coherence tended to be stronger in the pairs of guitarists who also showed high synchronization within brains (i.e., pairs 3, 4, and 7 in Figure S1).

### Synchronization after play onset

**Figure 2:**
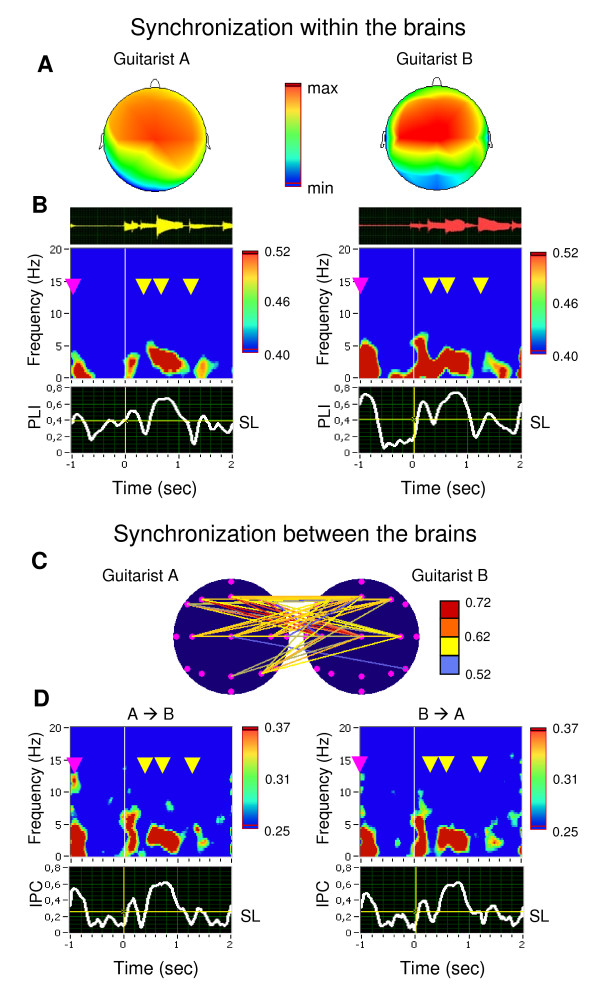
**Phase synchronization within and between the brains during the period of guitar playing**. (A) Topological distributions of PLI in a representative pair of guitarists, A and B, at the low theta frequency (3.3 Hz) 800 ms after play beginning of guitarist A. Fronto-central maxima of PLI are shown. (B) Guitar traces and time-frequency diagrams of average PLI for guitarists A and B. PLI was averaged across six fronto-central electrodes. Only significant PLI values (*p *< 0.01) are shown. Time zero is time locked to play onset of the leading guitarist A. The leading guitarist's finger gesture to start playing together is indicated with a red arrow. The yellow arrows refer to individual guitar strokes. The time course of PLI values at the low theta frequency (3.3 Hz) is depicted below the time-frequency diagram. (C) Interbrain synchronization between the two guitarists measured by IPC at the low theta frequency (3.3 Hz) 800 ms after play onset. Colored lines indicate synchrony between electrode pairs of the two guitarists. Only IPC values higher than 0.51 are highlighted. (D) Time-frequency diagram of the average IPC averaged across six electrode pairs (for further explanation, see Figure 1D and 2B). The time course of IPC values at the low theta frequency (3.3 Hz) is depicted below the time-frequency diagram. High phase synchronization within (PLI in 2B) and between (IPC in 2D) the brains took place not only at play onset but also at the time point of the gesture serving as starting signal, and at the individual guitar strokes. SL = significance level.

Synchronization within the brains during the time window of play onset was also highest over fronto-central sites (Figure 2A) but at a lower frequency range, that is, between 0.5 and 7.5 Hz with a maximum around 3.3 Hz (Figure 2B). Synchronization between brains in this case again primarily involved fronto-central connections (Figure 2C) and was also strongest in the frequency range between 0.5 and 7.5 Hz with a maximum around 3.3 Hz (Figure 2D). Interestingly, synchronization (within and also between the brains) was strongly related not only to play onset but also to the leading guitarist's starting gesture immediately prior to play onset, and to the onset of the starting note while playing (for details see Figure 2B, D). Here, interbrain synchronization was again higher in pairs who showed higher synchronization within brains (Figure S3 in Additional file 2).

**Figure 3:**
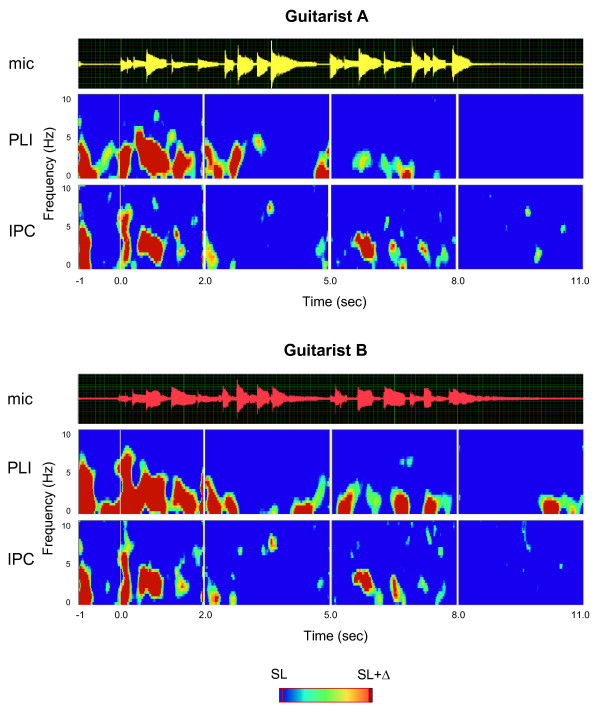
**Phase synchronization within and between the brains for the entire music sequence**. Acoustic guitar traces (mic) and time-frequency diagrams of average PLI and IPC for guitarists A and B. For analysis, the entire sequence was subdivided into four consecutive sections of equal length. Only significant PLI and IPC values are shown (*p *< 0.01). The overall significance level was set to the mean value across the four sections. Time is locked to the play onset of the leading guitarist (Guitarist A). In contrast to Figures 1 and 2, the time-frequency diagram is restricted to the frequency range of up to 10 Hz. SL = significance level; Δ = 0.12.

To examine synchronization during the playing of the entire piece, we analyzed three further 3s-sequences that were time-locked to the start of guitarist's A onset of play. Significance levels were first determined for each of the four 3s-sequences and then averaged across these sequences (Figure 3). Though synchronization both within and between the brains was considerably reduced relative to the first 2s after play onset, synchronization patterns in delta/theta frequency, especially in the time between the 5^th ^and 8^th ^seconds of the music piece, were found. These synchronization patterns were also related to the onset of the starting note while playing. Interestingly, in the time window between the 8^th ^and 11^th ^seconds, that is, after the end of play, between-brain synchronization disappeared completely.

### Relation between synchronization and behavioral measures

**Figure 4:**
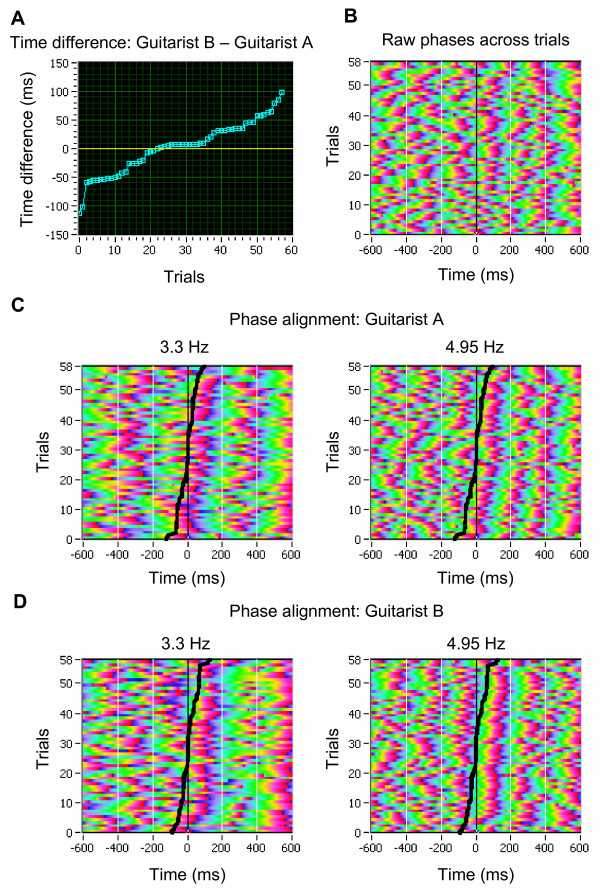
**Phase alignment of phase angles in single trials related to behavioral asynchrony of play onsets between the two guitarists**. (A) Asynchrony defined as the time difference (in ms) between play onsets of the two guitarists (guitarist B's play onset time minus guitarist A's play onset time) across 58 trials. (B) Time course of phase angles across trials (unsorted). (C) Phase alignment of phase angles at the two frequency bins (3.3 and 4.95 Hz, the second and the third harmonic of the metronome frequency) across trials in guitarist A. Trials were sorted by behavioral onset asynchrony between the players as shown in A. Behavioral asynchrony is depicted by the black curve. (D) Phase alignment of phase angles at the two frequency bins across trials in the guitarist B. Trials were again sorted by behavioral onset asynchrony between the players (guitarist A's play onset time minus guitarist B's play onset time), depicted by the black curve.

To test whether synchronization patterns were related to behavioral measures, we determined absolute phases in single trials and sorted them according to the time difference (asynchrony) between the play onsets of the two guitarists recorded through microphones. We computed this relationship for the two frequency bins that included the synchronization maxima, as mentioned above. We note that these frequencies happened to be identical to the second and the third harmonic of the metronome frequency (Figure 4). For each of the two guitarists the results indicated a strong phase alignment that closely followed behavioral onset asynchrony (for data of all other pairs, see Figure S4 in Additional file 2).

## Discussion

Synchronized brain activities within one brain have been observed before in relation to various tasks including music perception [29,35]. To the best of our knowledge, however, interbrain synchronization in general as well as within-brain synchronization during music production in particular has not been reported before. Synchronized theta (and delta) oscillations both within and between the brains were most pronounced when the musicians listened to the metronome to set their tempo and when they started playing a short melody together. Recently, increased brainwave synchronization at delta frequency measured by ITC (inter-trial coherence, a phase synchronization measure like PLI) was found as a response to periodic stimulation with slow repetition rates of 1–8 Hz [36]. Accumulating evidence indicates that behavior-dependent neuronal oscillations in the mammalian cortical network can be observed across a wide frequency range from approximately 0.05 to 500 Hz. Network oscillations may functionally bias stimulus selection, form transient binding of neuronal assemblies, and facilitate synaptic plasticity [37]. Oscillations in the theta frequency range are prominent in the human and animal EEG, with possible functional roles covering a wide spectrum of behaviors, ranging from orienting reflexes to conditioning, arousal, attention, learning, and memory binding mechanisms [38]. Other than perceptual and cognitive functions, it has been suggested that theta oscillations are also important for motor functions. For instance, Vanderwolf's "voluntary movement" hypothesis [38,39] suggests that theta rhythms support intentional movement. The findings of synchronized brain activities at the theta frequency both within and between brains lend support to this hypothesis, and extend it to interpersonally coordinated voluntary movements.

Beyond coordinating voluntary movements among adults, interbrain oscillatory couplings as observed here may also serve important functions in early social development (e.g., mother-child interaction) and for 'theory of mind' capabilities (i.e., the abilities to explain and predict other people's behavior by attributing to them independent mental states such as beliefs, intentions, emotions, and expectations) [1,4,15]. Given the relatively low spatial resolution of EEG, we can only speculate about the neuronal circuitry contributing to within-brain and interbrain synchronization during music production. Increased synchronization at frontal and central electrode sites may indicate coordinated firing of neuronal assemblies located in motor and somatosensory cortex, which control and coordinate motor activity. Furthermore, we conjecture that neural networks supporting social cognition, in general, and theory of mind abilities, in particular, might also support interbrain couplings during interpersonally coordinated voluntary actions. Neural activity in medial prefrontal cortex is selectively enhanced during theory of mind tasks [27,28]. Specifically, these increments may reflect synchronization of cell assemblies representing (i.e., mirroring) the coordinated behavior between oneself and others [2,3]. To further explore the social and developmental functions of interbrain coherence, future studies require measures and methods with higher spatial resolution and experimental designs that specifically aim at social perspective taking.

As predicted, frontal and central regions showed the strongest synchronization patterns within and between the brains. At the same time, as shown in Table 1 and in Figure S2, temporal and parietal regions also showed relatively high synchronization in at least half of the guitarist pairs (for details see Figure S2). Thus, activities in the temporal and parietal regions might also be involved in the processes supporting interpersonal action coordination and music production. There is evidence to suggest that these regions could be activated during music perception and also during music production [26] as well as through pleasant feelings induced by the music [40,41].

Another question arising here is to what extent interbrain synchronization observed during preparatory metronome tempo setting and coordinated play onset reflects 'genuine' inter-person interactions. Synchronizations were observed at low EEG frequencies, suggesting contributions from stimulus- and movement-related processes. During metronome tempo setting, both guitarists heard the same stimuli, that is, they shared the same sensory inputs. Therefore, it may not come as a surprise that they showed similar patterns of within-brain phase locking. While this "passive", stimulus-induced mechanism may have contributed to interbrain phase coherence during preparatory metronome tempo setting and coordinated play onset, we doubt whether it can fully explain the observed patterns of interbrain synchronization. As is to be seen in Figure S1, all pairs of guitarists showed strong within-brain synchronization practically to each metronome tap, but the extent of interbrain synchronization was considerably reduced and not always present, and also depended on the frequency in which the guitarists synchronized their brain potentials. The same observation holds for the coordinated play onset presented in Figures 2 and S3. As shown in Figure 3, synchronization both within and between the brains was strongest in the time period directly after play onset and was reduced thereafter, in other words, it followed an intrinsic dynamic, that was apparently related to the dynamic or structure of the musical piece itself (e.g., increased synchronization in the time between the 5^th ^and 8^th ^seconds of the piece, and decreasing synchronization thereafter). In addition, this synchronization was strongly related to behavioral measures of play onset asynchrony, as shown in Figures 4 and S4. Here, phase alignment in the two frequencies, which showed synchronization maxima, strongly followed the time onset differences between the two guitarists. Based on this evidence, we contend that the observed degree of between-brain synchronization cannot be reduced to processing similarities induced by attending to identical external stimuli, but also reflects the outcome of dynamic behavioral interactions between the two guitarists.

Identifying the brain mechanisms supporting interpersonal action coordination is exceedingly difficult. If two or more subjects share the same sensory inputs and produce similar motor outputs, interbrain synchronizations might arise because of similar neural responses to the shared sensory inputs and motor outputs, without necessarily reflecting neural processes of social interaction. To some extent, this quandary cannot be overcome because interbrain synchronization as a mechanism for interpersonal action coordination crucially depends on the presence of shared percepts, including the perception of the other person's actions (e.g., gesturing), or the perception of the product of these actions (e.g., sounds).

The present experiment has limitations and leaves room for questions to be addressed in future research. First, the sample size of our study was small. However, the main patterns of within- and between-brain synchronization replicated across all of the eight pairs of guitarists we investigated. Second, the synchronization measures used in this study referred to synchronization across trials. In future analyses, single-trial algorithms should be considered as well, as they allow investigating direct relations between synchronization indexes and performance parameters of interpersonal coordination. Third, the synchronization measures used in this study reflect only one aspect of phase synchronization, namely, 1:1 synchronization, or synchronization at a given frequency. Relative (i.e., n:m) synchronization as well as nonlinear (or weak) synchronization [34] may also be important for interbrain dynamics and should be investigated in the future. Fourth, we did not analyze the directionality of interbrain synchronization processes using asymmetric synchronization measures [42]. In conjunction with high-density behavioral assessments, such measures will shed further light on behavioral and neuronal mechanisms of interpersonal action coordination.

## Conclusion

This study demonstrates that data acquisition and analysis methods for simultaneous EEG recordings from multiple persons are important for discovering interbrain oscillatory couplings during interpersonal interactions. The results of the study show that interpersonally coordinated actions are preceded and accompanied by between-brain oscillatory couplings. Synchronization patterns during guitar playing assessed in terms of phase alignment after play onset were also related to behavioral play onset asynchrony (see Figures 4 and S4). Thus, patterns of interbrain synchronization reflect the temporal dynamics of interpersonal coordination. However, the present findings do not provide a firm answer to the question whether interbrain synchronization is causally linked to mechanisms of interpersonal action coordination, or whether it merely reflects the similarities in the percepts and movements of the interaction partners. Future research needs to examine more closely whether between-brain oscillatory couplings play a causal role in initiating and maintaining interpersonal action coordination.

## Methods

### Subjects

Nine pairs of professional guitarists participated in the study. One pair was excluded from data analysis because of recording artefacts. Analyses presented here are based on the remaining eight pairs of guitarists. In the first four pairs, the lead guitarist was always the same individual. In the remaining four pairs, the lead guitarist differed between pairs. Altogether, then, 13 guitarists participated in the study. Participants' mean age was 29.5 years (SD = 10.0). All participants played guitar professionally for more than 5 years (mean = 15.7 years, SD = 9.3). The Ethics Committee of Max Planck Institute for Human Development approved the study, and the study was performed in accordance with the ethical standards laid down in the 1964 Declaration of Helsinki. All subjects volunteered for this experiment and gave their written informed consent prior to their inclusion in the study.

### EEG Data Acquisition

EEG measurement took place in an acoustically and electromagnetically shielded cabin. In each trial, each pair of participants played a short melody (about 10–20 s) in unison. Data were collected across 60 trials. At each trial, before the duo started playing, at least four metronome beats were presented to the guitarists through a microphone connected to loudspeakers placed in the cabin. After the metronome beats, the leading guitarist (always guitarist A) signaled the beginning of playing together by tapping with the right finger on the guitar board. The sounds of the guitars were recorded through two microphones (i.e., one for each guitar) on two EEG channels, simultaneously with the EEG recordings. In addition, video and sound were recorded using Video Recorder Software (Brain Products, Munich, Germany) synchronized with EEG data acquisition. Microphone and video recordings were useful for determining the event triggers that later were set off-line in the EEG recordings. EEG was simultaneously recorded from both participants using two electrode caps with 64 Ag/AgCl electrodes placed according to the international 10–10 system, with the reference electrode placed at the right mastoid. For further analysis, data were re-referenced off-line to an average of the left and right mastoids. Separate amplifiers with separate grounds were used for each individual, optically coupled to a computer. Vertical and horizontal electrooculograms (EOGs) were recorded for control of eye blinks and eye movements. The sampling rate was 5000 Hz. Recorded frequency bands ranged from 0.01 to 1000 Hz. EEG recordings were filtered off-line with a band pass ranging from 0.5 to 70 Hz, and corrected for eye movements using the Gratton and Coles algorithm [43]. Eye-blink artifacts and artifacts from head and body movements were rejected based on the gradient criterion, that is, a maximum admissible voltage step (50 mV), and a difference criterion, that is, a maximum admissible absolute difference between two values in a segment (200 mV), and also by visual inspection.

### Data Analysis

For the analysis, event triggers were placed at the onset of the metronome beats and at play onset. Afterwards, EEG was resampled at 1000 Hz. Spontaneous EEG activity was divided into 3-s epochs related to the second metronome beat and play onset of the leading guitarist. Artefact-free epochs were analyzed using a complex Gabor expansion function, which transformed the EEG time series (1000 ms pre event- and 2000 ms after event onset) into a complex time-frequency signal *y*(*f*_*n*_, *t*) for frequencies up to 20 Hz. The components of the complex signal *y*(*f*_*n*_, *t*) – the Gabor coefficients – form a matrix of size *m *× *n*, where *m *is frequency (with a resolution of 0.33 Hz) and *n *is time (with a resolution of 1 ms). Two different synchronization measures were obtained from these complex time-frequency matrixes [44]: (i) the phase locking index (PLI), defined by

$$\begin{array}{cc} PLI(f_{n},t)=\left|\langle e^{j\cdot \Phi^{k}\left(f_{n},t\right)}\rangle \right|, & j=\sqrt{-1}, \end{array}$$

as a phase synchronization measure across the trials at the different electrodes within the brain, and (ii) the interbrain phase coherence (IPC), defined by

$$IPC_{\Phi }(f_{n},t)=\left|\langle e^{j\cdot \Delta \Phi^{k}\left(f_{n},t\right)}\rangle \right|,j=\sqrt{-1},$$

where the phase difference refers to two electrodes, one per brain/person, $\Delta \Phi^{k}=\mathrm{mod}\left(\Phi_{1}^{k}\left(f_{n},t\right)-\Phi_{2}^{k}\left(f_{n},t\right),2\cdot \pi \right)$, with instantaneous phases of these two electrodes across k trials $\Phi_{1}^{k}\left(f_{n},t\right)=\arg\left\{y_{1}^{k}\left(f_{n},t\right)\right\}$ and $\Phi_{2}^{k}\left(f_{n},t\right)=\arg\left\{y_{2}^{k}\left(f_{n},t\right)\right\}$.

Because the methods for determination of phase synchronization are time-consuming, we only selected 16 electrodes based on the 10–20 system (F7, F3, Fz, F4, F8, T7, C3, Cz, C4, T8, P7, P3, Pz, P4, P8, and Oz). We chose these electrodes because they are distributed across the entire cortex (10–20 system) so that the information of the remaining electrodes would be rather redundant in relation to the selected electrodes. To provide an overview of the observed synchronization measures, we determined the maximum PLI and IPC values for each of the 16 electrodes. During the preparatory period, the PLI and IPC peaks at the frequency of 4.95 Hz were determined across all four metronome beats within the whole 3-s period. During the guitar playing, the peaks at the frequency of 3.3 Hz were determined within the time period of 2 s after play onset. The maximum PLI and IPC values were then averaged across the 8 pairs of guitarists, and mean and standard deviation of these maxima were calculated. For time-frequency presentation, PLI values from six fronto-central electrodes as well as IPC values from six fronto-central electrode pairs (i.e., the Cz electrode of one guitarist to the six fronto-central electrodes of the other guitarist) were chosen and averaged in the time-frequency domain. Then, the averaged PLI and IPC values across all frequency bins within a 300-ms pre-stimulus interval were tested for normal distribution (Lilliefors test, α = 1%). Significance levels were set relative to the 300-ms pre-stimulus interval as the mean PLI or IPC values plus 3 standard deviations (*p *< 0.01). Time-frequency diagrams only display values above this threshold. Frontal and central electrodes were chosen for statistical analysis because they showed the largest synchronization effects (see Table 1 and in Figs. 1 and 2). When analyzing time-frequency diagrams for the entire musical piece, significance levels were first determined for 300-ms time intervals in each of the four 3-s epochs and then averaged across these epochs. In addition to analyses of PLI and IPC as described in the text, we also determined phase angles for each time-frequency pair, which can be extracted from the complex coefficients of the matrix. The phase angles were computed with respect to the 3-s epochs related to play onset of guitarist A and B, respectively. Using information about the phase angle, we computed phase alignment across trials for two frequency bins containing the synchronization maxima. The phase alignment based on each guitarist's data was then sorted as a function of the behavioral asynchrony in play onsets between the two guitarists.

## Authors' contributions

UL, S-CL and VM designed the study. WG programmed the Matlab scripts for data analysis. VM acquired and analyzed the data. UL, VM, and S-CL wrote the first draft of the manuscript. WG helped to draft the manuscript. All authors read and approved the final version of the manuscript.

## Supplementary Material

Additional file 1 **Supplemental movie. ** This movie shows the simultaneous video and EEG recordings of a pair of guitarists playing together for three trials. EEG from six channels (F3, Fz, F4, C3, Cz, C4) of both guitarist (a and b) as well as microphone recordings of guitar A (micA) and B (micB) are shown. The trial started with four metronome beats followed by the leading guitarist's starting gesture (can be seen also in the micA channel) immediately prior to play onset. Click here for file

Additional file 2 **Supplemental data. **Four Figures (S1, S2, S3, and S4) with corresponding legends are contained within the file. The Figures show the effects described in the paper in all eight guitarists pairs investigated in the study. Click here for file
