# Supplementary material for: Brains swinging in concert: cortical phase synchronization while playing guitar
Source: BMC Neurosci. 2009 Mar 17;10:22. doi: 10.1186/1471-2202-10-22 (PMC2662862; doi:10.1186/1471-2202-10-22)
Supplement: Additional file 2 — Supplemental data. Four Figures (S1, S2, S3, and S4) with corresponding legends are contained within the file. The Figures show the effects described in the paper in all eight guitarists pairs investigated in the study. [file 1471-2202-10-22-S2.pdf]

Supplemental Data

Brains swinging in concert: Cortical phase synchronization while playing guitar

Ulman Lindenberger, Shu-Chen Li, Walter Gruber, and Viktor Müller

Figure S1. Phase synchronization within and between the brains during the preparatory period of metronome tempo setting in the eight guitarists pairs (P1-P8). **(A)** Time-frequency diagram of average PLI for guitarist A and B separately. PLI was averaged across six fronto-central electrodes (F3, Fz, F4, C3, Cz, and C4). Only significant PLI-values ($p < 0.01$) are highlighted. **(B)** Time-frequency diagram of the average IPC averaged across six electrode pairs. On the left diagram (A -> B), the selected electrode pairs represent phase coherence between one electrode of guitarist A (Cz) to the six fronto-central electrodes of guitarist B (F3, Fz, F4, C3, Cz, and C4). On the right diagram (B -> A), the selected electrode pairs represent phase coherence between one electrode of guitarist B (Cz) to the six fronto-central electrodes of guitarist A. Only significant IPC-values ($p < 0.01$) are highlighted. Time zero is time locked to the second metronome beat. Metronome beats are depicted with white arrows. SL = Significance Level; $\Delta = 0.12$.

Figure S2. Phase synchronization peaks during preparatory period of metronome tempo setting and during period of guitar playing. Synchronization within (PLI maxima) and between (IPC maxima) the brains across the 16 electrodes in the 8 pairs of guitarists are presented as radar plots (lines in different colours indicate different pairs). All the axes are ranged between 0 indicating no synchronization (at the center of the radar plot) and 1 indicating highest synchronization (at the extremes of the radar plot).

Figure S3. Phase synchronization within and between the brains during the period of guitar playing in the eight guitarists pairs (P1-P8). **(A)** Time-frequency diagram of average PLI for guitarist A and B separately. PLI was averaged across six fronto-central electrodes (F3, Fz, F4, C3, Cz, and C4). Only significant PLI-values ($p < 0.01$) are highlighted. **(B)** Time-frequency diagram of the average IPC averaged across six electrode pairs. On the left diagram (A -> B), the selected electrode pairs represent phase coherence between one electrode of guitarist A (Cz) to the six fronto-central electrodes of guitarist B (F3, Fz, F4, C3, Cz, and C4). On the right diagram (B -> A), the selected electrode pairs represent phase coherence between one electrode of guitarist B (Cz) to the six fronto-central electrodes of guitarist A. Only significant IPC-values ($p < 0.01$) are highlighted. Time zero is time locked to play onset of the leading guitarist. The leading guitarist's finger gesture to start playing together is depicted with the red arrow. The yellow arrows marked single guitar strokes as recorded. SL = Significance Level; $\Delta = 0.12$.

Figure S4. Phase alignment of phase angles in single trials related to behavioural asynchrony of play onsets between the two guitarists in the eight guitarists pairs (P1-P8). **(A)** Phase alignment of phase angles at the two frequency bins (3.3 and 4.95 Hz) across trials in guitarist A. Trials were sorted by behavioural onset asynchrony (depicted by the black curve) between the players (guitarist B's play onset time minus guitarist A's play onset time). **(B)** Phase alignment of phase angles at the two frequency bins (3.3 and 4.95 Hz) across trials in guitarist B. Trials were sorted by behavioural onset asynchrony (depicted by the black curve) between the players (guitarist A's play onset time minus guitarist B's play onset time).

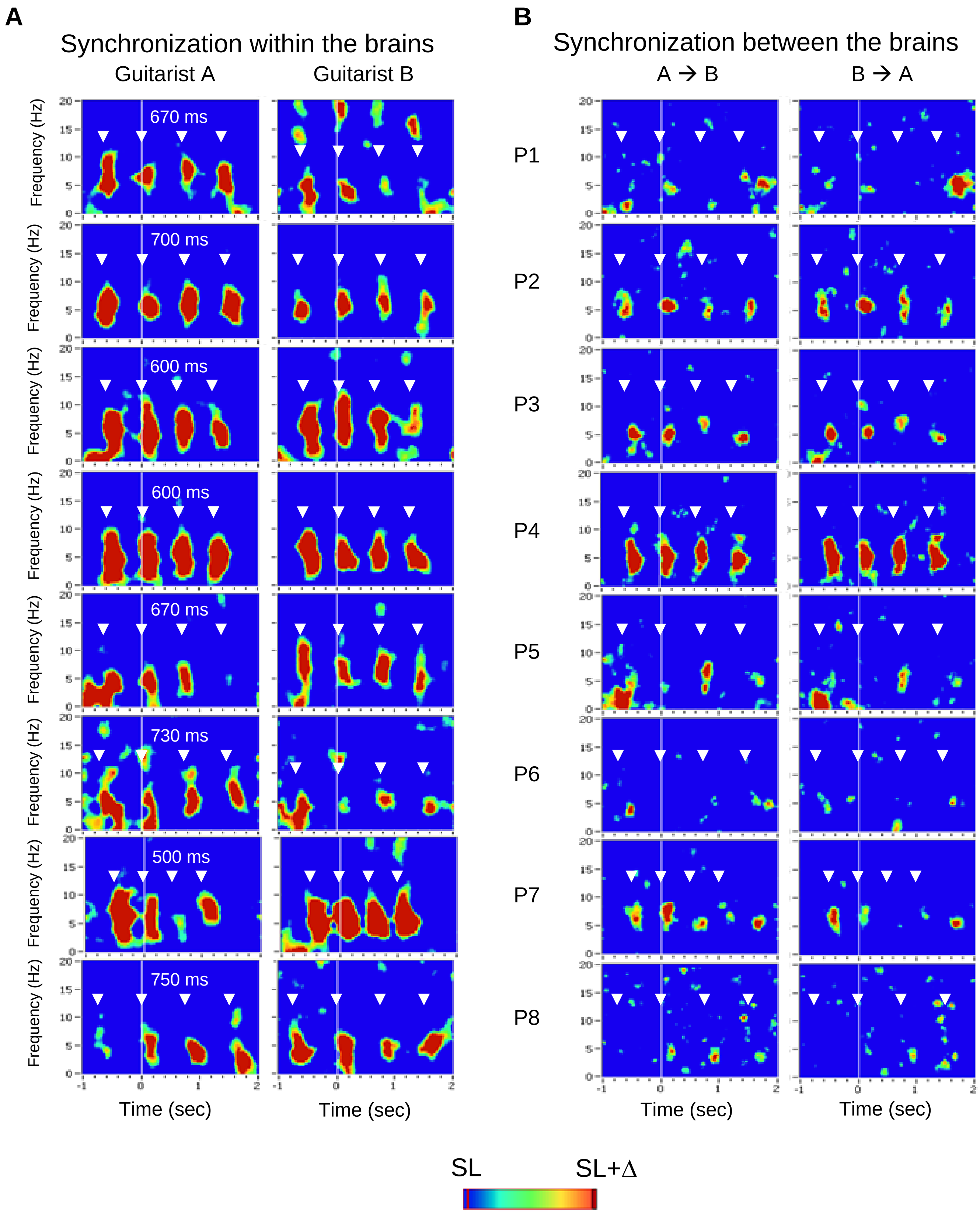
A
Synchronization within the brains
Guitarist A
Guitarist B
670 ms
700 ms
600 ms
600 ms
670 ms
730 ms
500 ms
750 ms
Frequency (Hz)
Time (sec)
B
Synchronization between the brains
A → B
B → A
P1
P2
P3
P4
P5
P6
P7
P8
Time (sec)
SL
SL+Δ

Preparatory period of metronome tempo setting

Synchronization within the brains

Guitarist A

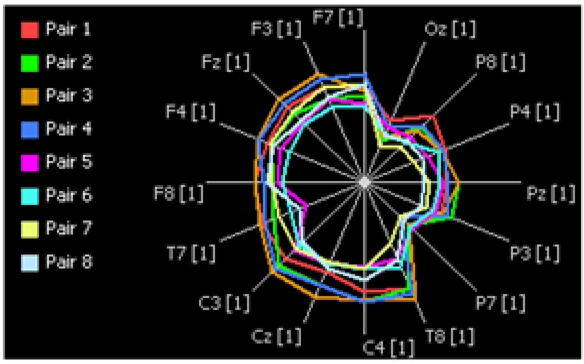

Guitarist B

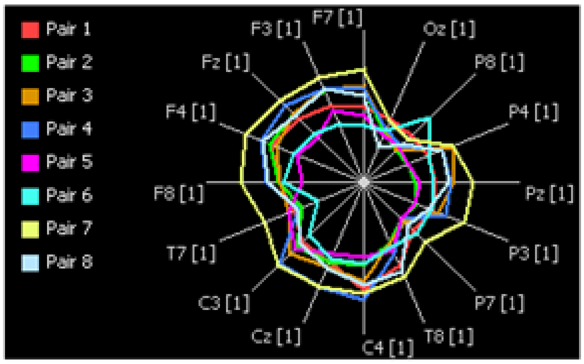

Synchronization between the brains

A -> B

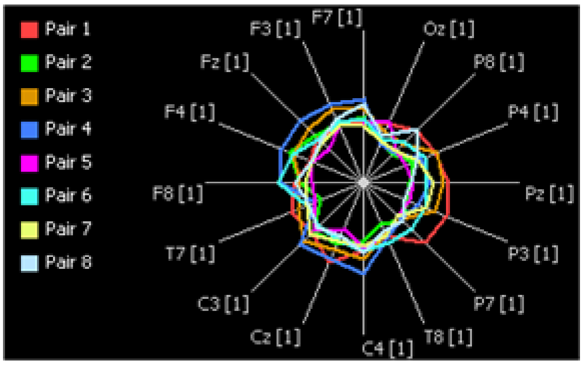

B -> A

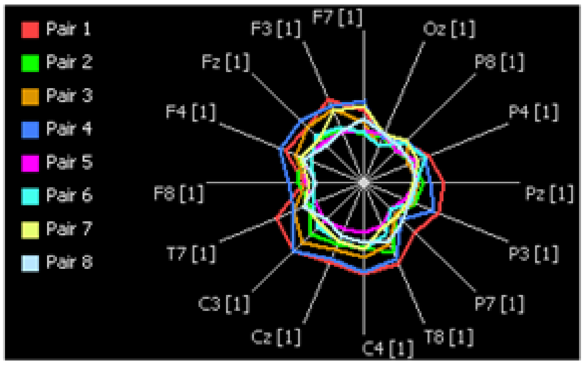

Period of guitar playing

Synchronization within the brains

Guitarist A

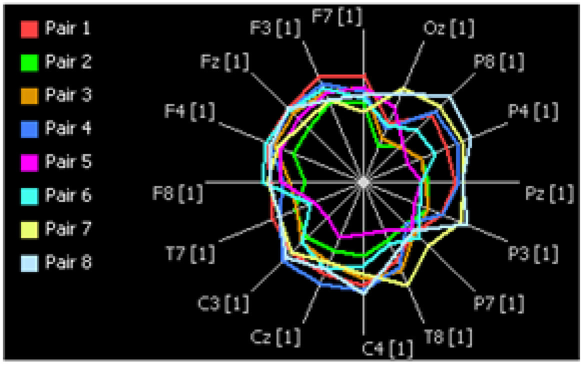

Guitarist B

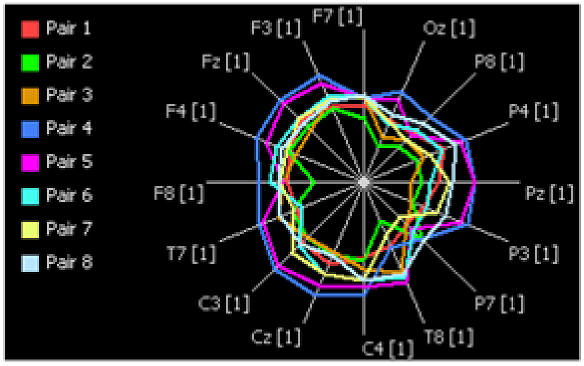

Synchronization between the brains

A -> B

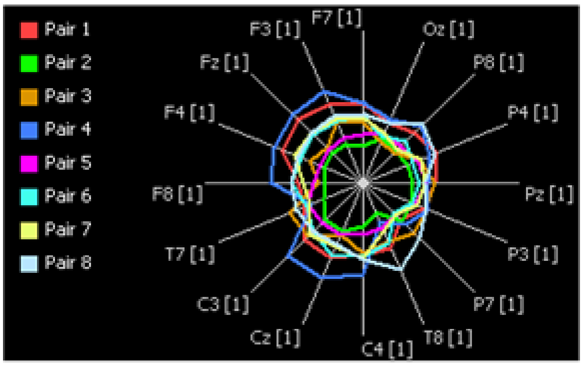

B -> A

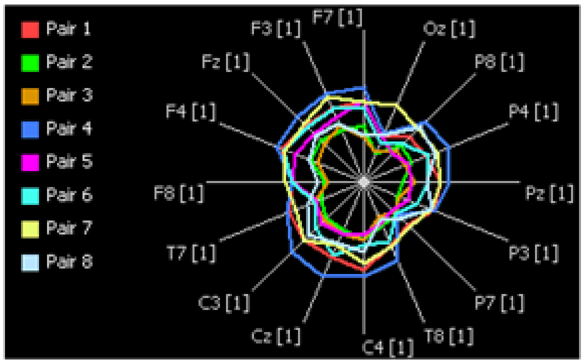

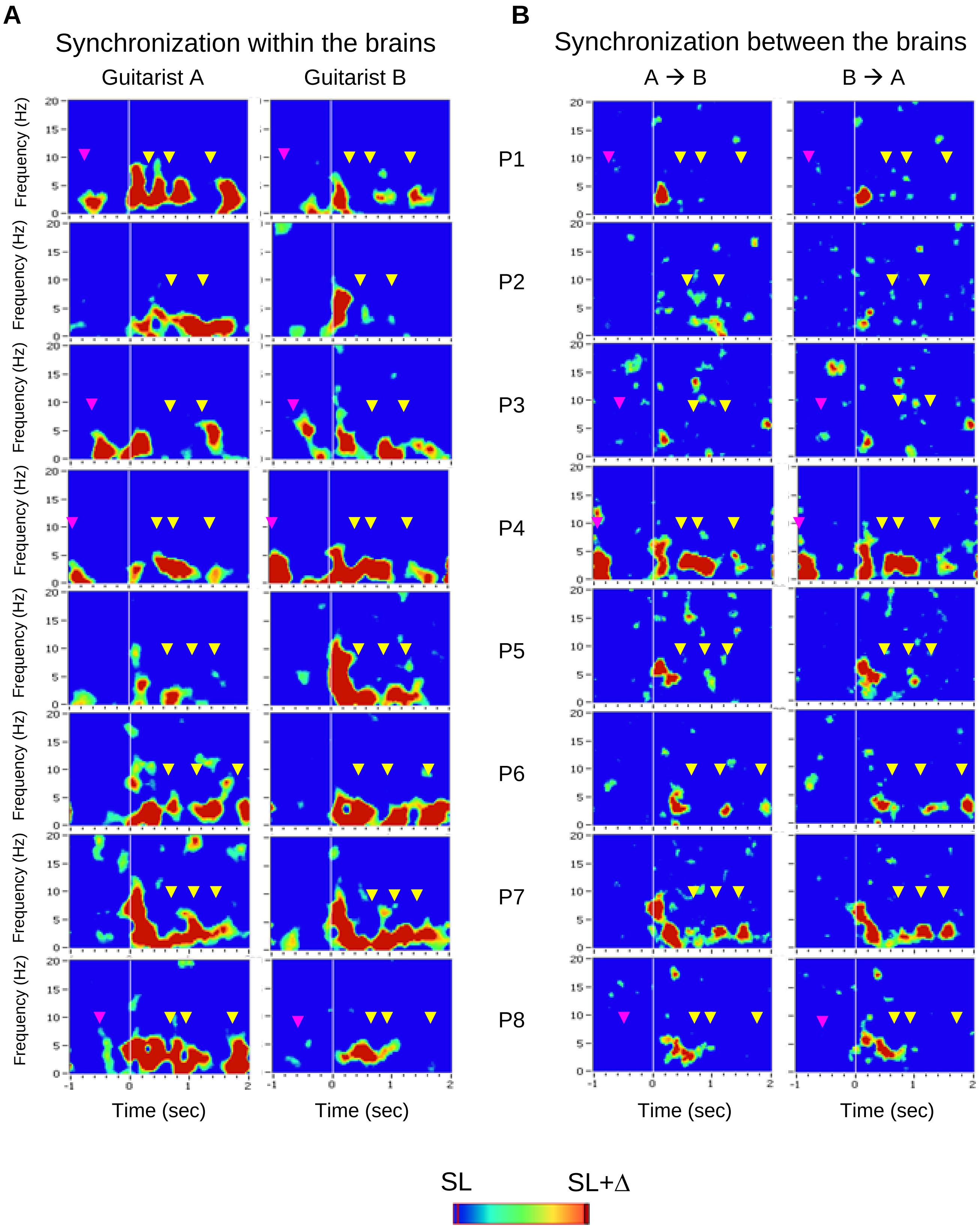

A
Synchronization within the brains
Guitarist A
Guitarist B
B
Synchronization between the brains
A → B
B → A
P1
P2
P3
P4
P5
P6
P7
P8
Frequency (Hz)
Time (sec)
SL
SL+Δ

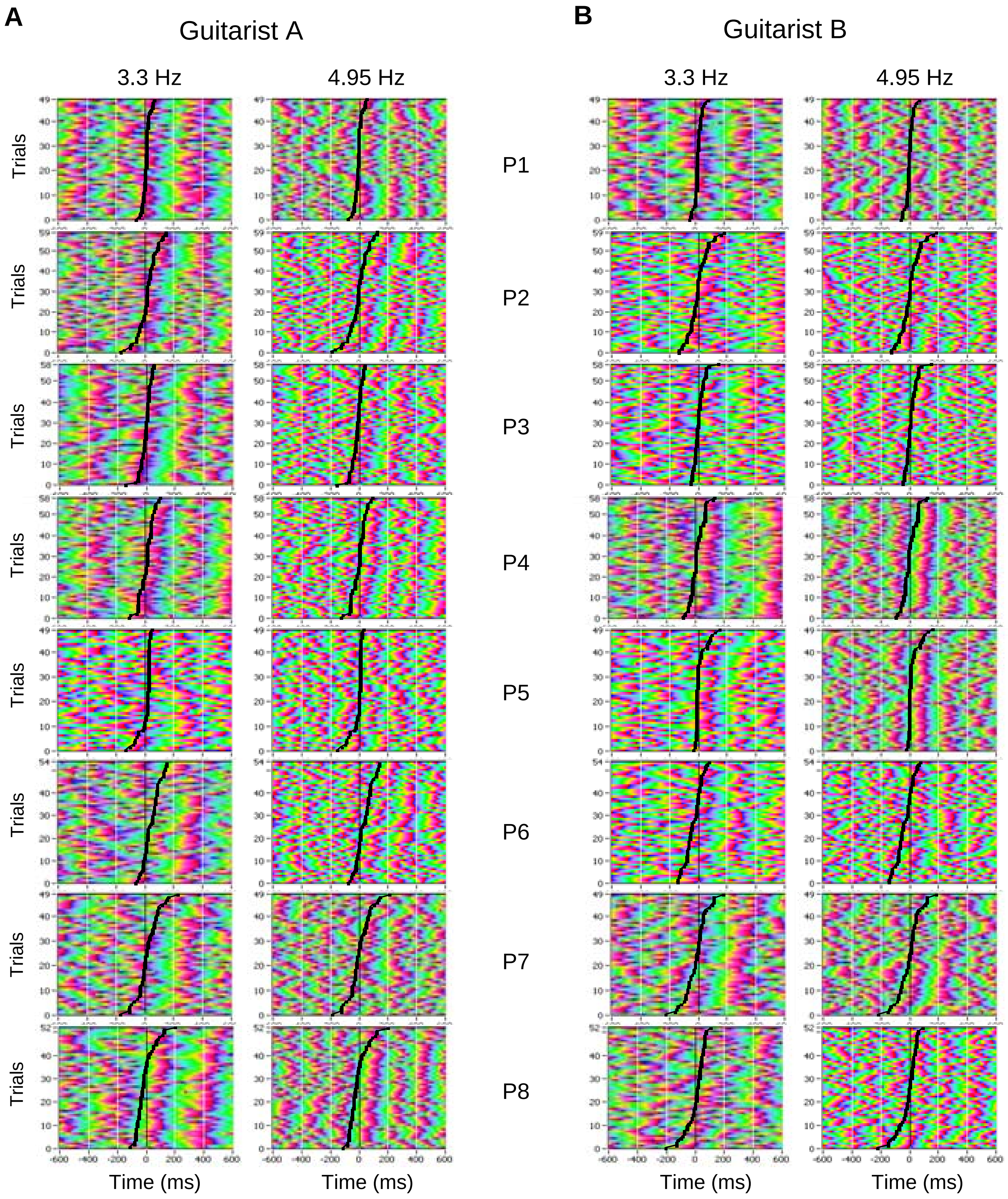
A
Guitarist A
B
Guitarist B
3.3 Hz
4.95 Hz
3.3 Hz
4.95 Hz
P1
P2
P3
P4
P5
P6
P7
P8
Trials
Time (ms)
Time (ms)
Time (ms)
Time (ms)
